# The nociceptive withdrawal reflex during spinal analgesia in pigs undergoing veno-arterial extracorporeal membrane oxygenation: a prospective observational study

**DOI:** 10.3389/fvets.2024.1449297

**Published:** 2024-12-11

**Authors:** Mariafrancesca Petrucci, Claudia Spadavecchia, Kaspar F. Bachmann, David Berger, Alessandro Mirra, Daniela Casoni

**Affiliations:** ^1^Experimental Surgery Facility, Experimental Animal Center, Faculty of Medicine, University of Bern, Bern, Switzerland; ^2^Department for BioMedical Research, Faculty of Medicine, University of Bern, Bern, Switzerland; ^3^Anaesthesiology and Pain Therapy Section, Department of Clinical Veterinary Sciences, Vetsuisse Faculty, University of Bern, Bern, Switzerland; ^4^Department of Intensive Care Medicine, Inselspital, Bern University Hospital, University of Bern, Bern, Switzerland

**Keywords:** NWR, neuraxial analgesia, nociception, swine, ropivacaine, morphine, VA-ECMOe

## Abstract

**Introduction:**

Use of veno-arterial extracorporeal membrane oxygenation (VA-ECMO) is still in the focus of research, in which pigs are commonly involved. During VA-ECMO, cardiovascular parameters are artificially manipulated and therefore not reliable indicators of nociception. Nociceptive withdrawal reflex (NWR) thresholds can be a suitable alternative in such a context. This study aimed at recording and comparing NWR thresholds before and after administering spinal analgesia in healthy pigs undergoing VA-ECMO.

**Methods:**

Sixteen pigs were sedated with a mixture of ketamine, midazolam, and methadone; general anesthesia was induced with propofol and maintained with propofol and fentanyl in continuous rate infusion. Before surgery, ropivacaine 0.75% and morphine (RM) were injected via a spinal catheter (T13-L1). Nociceptive withdrawal reflex thresholds were recorded before RM (baseline) and at 40 min, end of surgery, 240, 300, 360, 420 and 480 min afterward. If after spinal analgesia NWR thresholds increased ≥20% from their baseline values, the increase was deemed clinically relevant. If NWR thresholds decreased at least 20% from their baseline values, ropivacaine alone was injected (rescue analgesia). Thresholds were compared with baseline using ANOVA on Ranks followed by Dunn’s method. At each time point, the number of pigs showing a clinically relevant increase in thresholds, thresholds higher than the maximum stimulation intensity and the need of rescue analgesia, was assessed. Nine animals were included in the final data analysis.

**Results:**

A clinically relevant increase of the thresholds was achieved in all the pigs at 240 min after the injection of RM. A statistically significant increase in NWR thresholds was found at 300 and 360 min (*p* = 0.009 and 0.048, respectively) compared to baseline. Rescue analgesia was required at 300 (one pig) and 420 (two pigs) and 480 (one pig) minutes.

**Discussion and conclusion:**

Nociceptive withdrawal reflex thresholds increased significantly, both clinically and statistically following spinal injection. Their increase suggests that the combination of spinal morphine and ropivacaine can last on average up to 6 h. Particularly in those scenarios where cardiovascular variables are unreliable, NWR thresholds could be useful for evaluating antinociception following spinal analgesia in pigs.

## 1 Introduction

Extracorporeal membrane oxygenation (ECMO) is a rescue therapy for patients who experience severe pulmonary and cardiac dysfunction (1). It supports the function of the heart and the lungs and provides adequate blood flow to the organs (2). It may be used during high-risk cardiac, thoracic or trauma surgeries (3) and during cardio-pulmonary resuscitation (4).

As clinical application of ECMO and weaning strategies (i.e., discontinuation of ECMO support) are still under investigation, pigs are commonly used as animal model due to their anatomical and physiological similarities to humans (5–7). If central ECMO is investigated, the heart must be accessed to establish cannulation of the ascending aorta and the right atrium. Therefore, sternotomy is required, which is recognized to be particularly painful, both intra and post-operatively (8). A correct assessment and treatment of nociception in animal experimentation must be guaranteed for both ethical and legal reasons. Therefore, the adoption of a solid and efficacious antinociceptive and analgesic strategy is required (8, 9). While the use of spinal analgesia is highly debated in cardiac surgery due to inherent risks associated with anticoagulation, it may be the preferred choice in experimental settings with terminal anesthesia, as risks related to the post-operative period (e.g., epidural hematoma) have not to be accounted for (10). In these cases, the ability of neuraxial anesthesia to attenuate the stress response to surgery (11) may overcome the potential side effects and provide hemodynamic stability. This is pivotal importance to avoid uncontrolled cardiovascular modifications associated with nociceptive stimuli, which would act as confounders.

Ropivacaine, an amido-amide local anesthetic, has been shown to provide adequate antinociception, cardiovascular stability and to reduce opioid consumption in major surgeries when administered epidurally (12). Moreover, when combined with *μ* opioids for both spinal and epidural administration, its antinociceptive activity is enhanced (12–15). While the analgesia duration of ropivacaine 0.75% alone can be up to 6 h when administered spinally in humans (16), to the authors’ knowledge, no information is available in pigs.

Nociception under general anesthesia is commonly assessed through the evaluation of cardio-respiratory parameters in both humans and veterinary species (17, 18). In pigs undergoing isoflurane anesthesia, mean arterial pressure (MAP) variation has been shown to be superior to other variables as indicator of nociception (19). However, as the extracorporeal flow is a major determinant of MAP during veno-arterial extracorporeal membrane oxygenation (VA-ECMO), potential modifications in the sympathetic system are not mirrored, and MAP is not helpful in evaluating the adequacy of the antinociception.

The nociceptive withdrawal reflex (NWR) is a polysynaptic spinal reflex, which results in the withdrawal of an area of the body in response to a potential tissue damage, with the aim to prevent it (20). The afferent section of the reflex arc is formed by Aδ and C fibers, which, when stimulated by a nociceptive stimulus, transmit it to the spinal cord. The efferent branch of the reflex is formed by motor neurons, which, once activated, generate a contraction of corresponding muscles. The NWR can be experimentally elicited by transcutaneous electrical stimulation of a sensory peripheral nerve and the electromyographic response from the involved muscles can be recorded and assessed (21). This technique has already been investigated in several animal species (22–24), including pigs (25), to test analgesic and anesthetic drugs’ ability to modify nociceptive thresholds (26, 27). No reports concerning NWR thresholds in pigs receiving spinal analgesia under general anesthesia have been published so far.

The aim of this study was to define and compare the NWR thresholds before and after the administration of spinal analgesia in pigs undergoing VA-ECMO. We hypothesized that the NWR thresholds would increase following spinal analgesia. Additionally, we hypothesized that tracking thresholds over time could provide insights into the duration of spinal analgesia.

## 2 Materials and methods

### 2.1 Animals

Sixteen pigs (*Suis scrofa domesticus*, Schweizer Edelschwein), six males and ten females, aged 14.5 ± 1.5 weeks, weighted 44.6 ± 3.2 kg were enrolled. These animals were purchased for an experimental study aiming to estimate pulmonary blood flow and right ventricular function in VA-ECMO using a modified Fick principle of thermodilution technique (5, 28). Animals could be included in the study if they showed a normal growth curve and normal appetite, and if they had no history of coughing, fever, diarrhea and antibiotic therapy in the previous 15 days. Pigs were transported 72 h prior to anesthesia from the farm of origin to the Vetsuisse Faculty, University of Bern, where they were housed in single boxes (1.45 m^2^) enriched with straw bedding and ropes. A light/dark cycle of 12 h was set, and maintenance of visual, olfactory, and auditory contact with co-mates was always guaranteed. Pigs were fed twice per day with *ad libitum* access to water. Food but not water was withdrawn 12 h before general anesthesia. The study was reviewed and approved by the Committee for Animal Experiments of the Canton of Bern, Switzerland (permission number: BE 111/18). For reporting all the performed procedures, the ARRIVE guidelines (Animals in Research: Reporting of *In Vivo* Experiments) were followed.

### 2.2 Anesthetic protocol

After clinical examination and American Society of Anesthesiology (ASA) physical status classification (16 pigs ASA 1), 15 mg/kg ketamine (Narketan, Vetoquinol AG, Bern, Switzerland), 0.5 mg/kg midazolam (Dormicum; Roche CH, Switzerland) and 0.2 mg/kg methadone (Methadon Streuli; Streuli Pharma AG, Switzerland) were mixed in the same syringe and injected intramuscularly at the level of the cleido-occipital muscle. The pigs were left undisturbed for 15 min and then lifted on a table for preparation and disinfection once sedation was deemed adequate. Oxygen was supplemented through a non-tight face mask (4–6 liters/min) until tracheal intubation was achieved. Pulse rate (PR) and oxygen saturation (SpO_2_) were monitored continuously through a pulse-oximeter probe placed on the lips. A venous catheter (20 Gauge, BD Ventflon Pro Safety, Switzerland) was placed in the auricular vein, fluid therapy (Ringer lactate’s solution) was started at 5 mL/kg/h and induction of general anesthesia was achieved with intravenous (IV) administration of propofol (Propofol 10 mg/mL, Fresenius Kabi, Switzerland) 1–4 mg/kg to effect. After endotracheal intubation, anesthesia was maintained with continuous rate infusion of propofol started at 150–200 μg/kg/min and fentanyl (5–10 μg/kg/h), and the endotracheal tube was connected to a circle breathing system. Pigs were mechanically ventilated in volume-controlled mode setting tidal volume (TV) at 10 mL/kg, positive end expiratory pressure (PEEP) at 5 cmH_2_O and fraction of inspired oxygen (FiO_2_) at 60% (C6 Ventilator, Hamilton Medical, Bonaduz, Switzerland). An arterial catheter (20 Gauge) was then placed in the coccygeal artery to monitor invasive blood pressure. During the entire duration of anesthesia, SpO_2_, invasive blood pressure, central venous pressure, electrocardiogram, esophageal temperature, inhaled and exhaled carbon dioxide (CO_2_, mmHg) and oxygen (O_2_) inspired fraction (%) level, palpebral reflex, eye position, jaw tone, electroencephalogram and bispectral index were continuously monitored (GE Healthcare Carescape B850, Anandic Medical System, Switzerland) and recorded every 5 min on an anesthetic sheet. Before sternotomy, amiodarone (Cordarone 150 mg/3 mL, Sanofi Aventis, Switzerland) 150 mg was administered IV over 30 min to prevent occurrence of lethal arrythmias. Noradrenaline (Noradrenaline 1 mg/mL, Sintetica AG, Switzerland) 0.05–0.1 μg/kg/min and adrenaline (Adrenalin 1 mg/mL, Sintetica AG, Switzerland) 0.1–0.2 μg/kg/min continuous rate infusion were administered to effect to maintain a mean arterial pressure (MAP) above 60 mmHg, when necessary. The infusion rate of propofol was titrated to a minimum of 100 μg/kg/h over time when burst suppressions were repeatedly noticed.

### 2.3 Nociceptive withdrawal reflex (NWR)

Nociceptive withdrawal reflex thresholds were obtained through the Pain Tracker device (Dolosys GmbH, Germany).

Once in the operating theater, skin preparation of the right hindlimb was performed to place the self-adhesive surface electrodes. Hair was clipped and shaved, and the skin was treated with abrasive tape, designed to remove the non-conductive skin layer, to achieve a better trace quality. The skin was then cleansed with betadine and alcohol, and carefully dried. Thereafter, stimulating (self-adhesive surface electrodes) and recording electrodes (subdermal needle electrodes) were positioned over the nerve digitalis plantaris and over the muscle tibialis cranialis, respectively. If the machine reported a good impedance value, the electrodes were secured in place.

Nociceptive withdrawal reflex was assessed through an established validated threshold tracking algorithm using transcutaneous electrical stimulation. The time window considered for analysis of the electromyographic response (EMG) (nociceptive reflex) was set between 80 and 240 milliseconds. Stimuli were delivered with 5 constant current square-wave pulses, with a duration of 1 millisecond and a frequency of 200 Hertz. The pain tracker monitor showed the real time NWR thresholds and their trend on the screen and stored the dataset on an external storage device. Moreover, thresholds were recorded on an on-purpose made experimental sheet.

### 2.4 Surgical procedure

The surgical procedure has been previously described and published (5). Briefly, lidocaine 1% (2 mg/kg) was injected before incision of the ventral cervical region; central venous catheters were then placed in both external jugular veins, and an arterial line was placed in the carotid artery. Afterwards, pigs underwent thoracotomy for setting up central VA-ECMO. Medial sternotomy was performed, and pericardium was opened. Thoracic vessels were then prepared, and, after administration of 80 IE/kg sodium heparin, the right atrium and ascending aorta were cannulated and connected to an ECMO circuit (Stockert SCPC Centrifugal Pump, Germany & Capiox FX15 Oxygenator, Terumo, USA). At the end of the procedure, thoracic cavity and pericardium were closed. During the whole duration of the surgery, activated clotting time was maintained between 180 and 220 s (3 times baseline) with a titrated infusion of heparin.

### 2.5 Spinal analgesia

To place a spinal catheter, the pig was positioned in sternal recumbency with the hindlimbs hyper flexed. An 18 Gauge (G) Tuohy needle was inserted on the median line, at the level of the intervertebral space between the last lumbar and the first sacral vertebra. Correct positioning of the needle was assessed by the presence of cerebrospinal fluid in the hub of the needle. Thereafter, the spinal catheter was introduced through the Tuohy needle and advanced to the thoraco-lumbar junction (T13-L1). The position of the tip of the catheter was verified by fluoroscopy.

Spinal analgesia was provided before starting the surgery of the cervical region, with 0.75 mg/kg ropivacaine 0.75% (Ropivacaine Fresenius 7.5 mg/mL, Fresenius Kabi AG, Switzerland) and 0.1 mg/kg morphine (Morphine HCl 2 mg/mL, Sintetica AG, Switzerland) up to a total volume of 0.1 mL/kg. The drugs were administered through the spinal catheter (Espocan, B Braun Medical AG, Switzerland) and the catheter flushed with 2 mL NaCl 0.9%.

### 2.6 Experimental protocol

**Figure 1:**
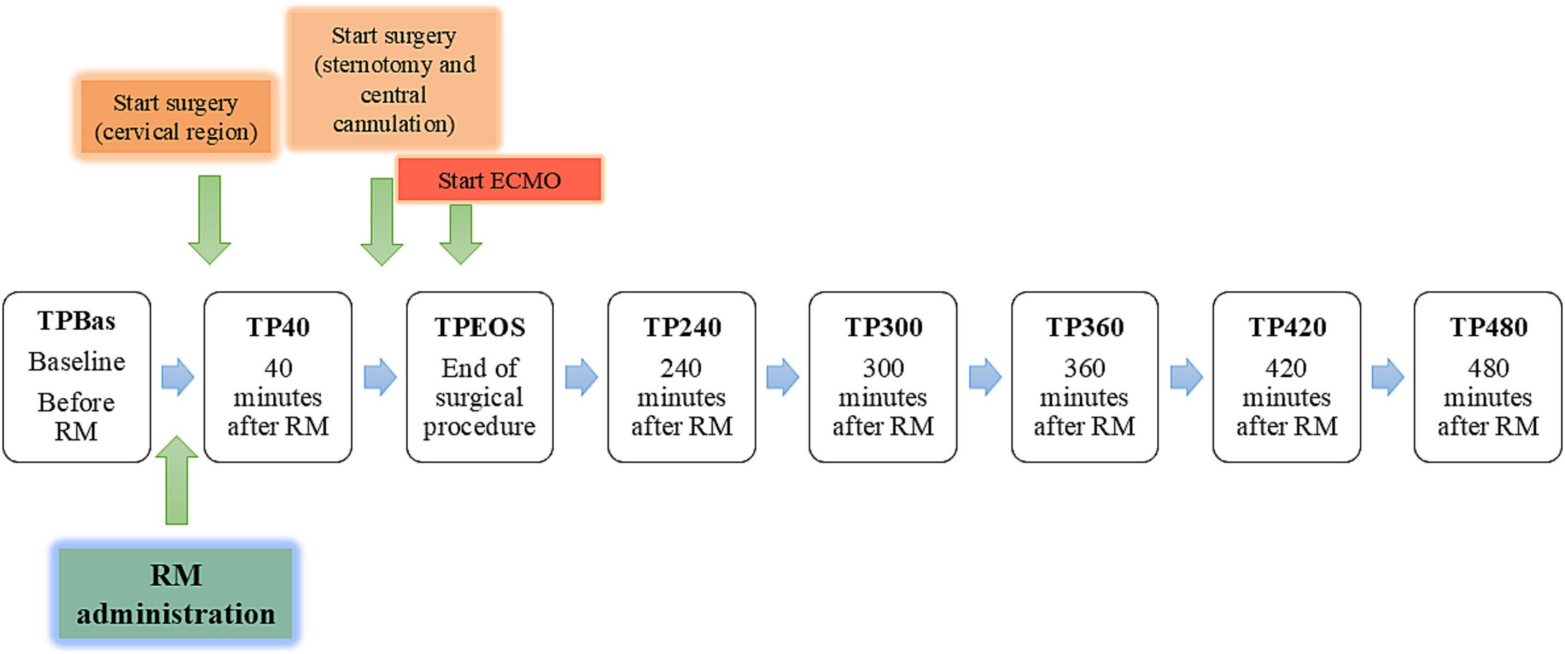
Graphic representation of the different time points (TPs) related to the administration of ropivacaine and morphine (RM). ECMO: extracorporeal membrane oxygenation.

Nociceptive withdrawal reflex thresholds were measured at different time points (TP) before and after spinal administration of ropivacaine and morphine (RM), as depicted in Figure 1.

If NWR thresholds showed an increase ≥20% from the baseline values, the increase was deemed clinically relevant. Conversely, if thresholds showed a decrease ≥20% from their baseline, rescue analgesia was provided with administration of 0.75 mg/kg ropivacaine 0.75% in the spinal catheter.

Heart rate (HR) and mean arterial pressure (MAP) were not influenced by VA-ECMO until TP40. Surgical procedures (placement of catheters in the cervical region and thoracotomy) started immediately after RM and lasted until TPEOS. Following TPEOS, pigs were maintained on VA-ECMO until the end of the trial.

During the whole study, at each TP and after determination of the NWR threshold, nociceptive flexor response to claw pinching and palpebral reflex were evaluated. Claw pinching was performed with a surgical clamp on the second or third hind toe (alternated) of the left hindlimb, connected to a spring balance, pulled until 60 Newton was reached and hold for 15 s. Pigs’ response to claw pinching was recorded as positive in case of withdrawal of the limb. Palpebral reflex was recorded as positive (if present) or negative (if absent).

### 2.7 Data analysis

Statistical analysis was performed with SigmaStat 4.0 (Point Richmond; CA, USA) and R Studio Statistical Software (version 4.3.2; R Foundation for Statistical Computing, Austria).

For each TP, NWR thresholds recorded by the Pain Tracker device over 1 min were averaged and used for statistical analysis. Data recorded and stored by the device were the first source of data retrieving, whereas, if the electronic storage failed, thresholds were retrieved from the experimental sheet. When recorded threshold were higher than the maximum stimulation intensity delivered by the device (150 mA), the value of 153 mA (the next step in the algorithm of measures) was considered for analysis. The thresholds recorded after rescue analgesia administration were not included in the statistical analysis.

Normality of the data was assessed with the Shapiro–Wilk test. Data are presented as mean ± standard deviation (SD) if normally distributed, and as median and interquartile range (IQR) [25th; 75th] if not normally distributed.

Thresholds were compared with baseline (TPBas) using ANOVA on Ranks test followed by Dunn’s method (non-parametric test). Moreover, at each TP, the number of pigs showing (a) a clinically relevant increase in thresholds, (b) thresholds higher than the maximum stimulation intensity and (c) the need of rescue analgesia was calculated.

The time between sedation and RM, and between induction of general anesthesia and RM was calculated.

Values of HR and MAP at TP1 and TP2 were compared using the Paired T-Test (parametric test) and Wilcoxon Signed Rank test (non-parametric test), respectively.

A two-tailed *p*-value ≤0.05 was considered statistically significant.

## 3 Results

**Figure 2:**
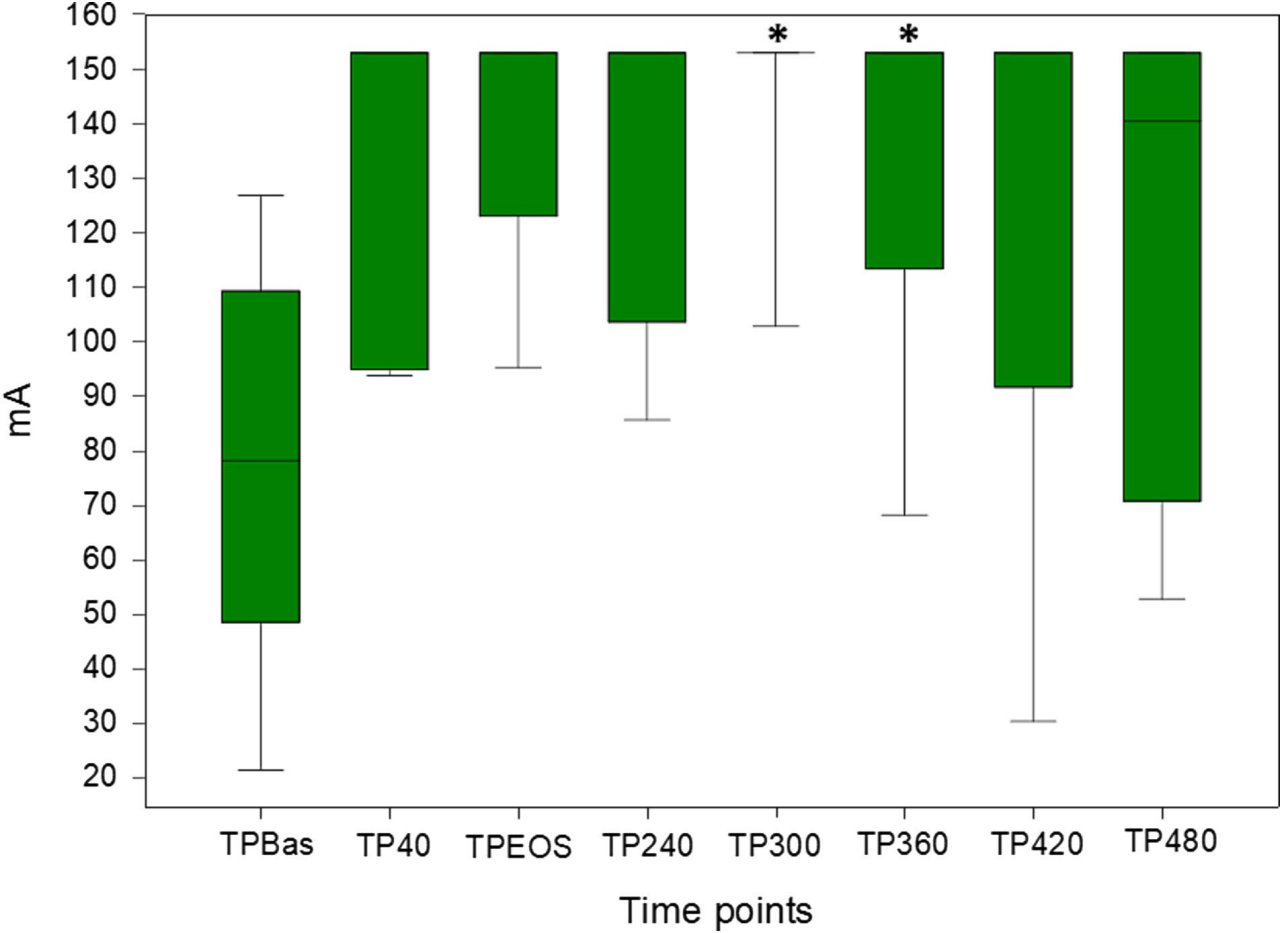
Box plots of nociceptive withdrawal reflex thresholds recorded at the different time points following spinal analgesia administration (ropivacaine and morphine: RM). Values reported as median and interquartile range [25th; 75th]. *statistically significant difference. mA: milliampere.

**Figure 3:**
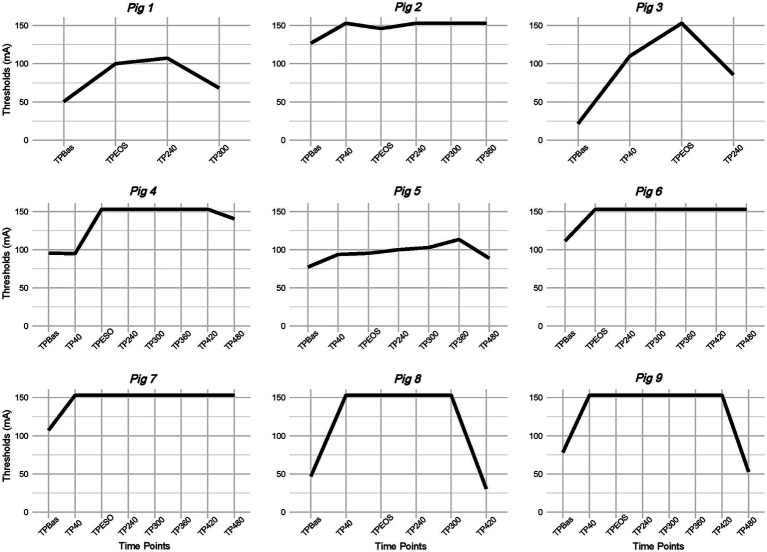
Nociceptive withdrawal reflex (NWR) thresholds modification across time points (TP) for each pig. mA: milliampere.

Nine pigs were included in the final data analysis. The first three pigs were used as pilot animals for feasibility assessment and method refinement, while four pigs were excluded due to absence of baseline measurement (*n* = 3) and missed fluoroscopic control of the spinal catheter (*n* = 1). Median and IQR [25th; 75th] of NWR thresholds recorded at different TPs following RM are reported in Table 1 and Figure 2. Due to reason related to the main experiment some measurements were not performed and details are reported in Figure 3. Due to device error, measurements from TP300 on are not reported in pig 3. Thresholds were retrieved by the experimental sheet six times (in three pigs at TP2, one at TP240, one at TP300, and one at TP420). The final number of animals in which NWR thresholds were recorded at each TP is reported in Table 1.

**Table 1:** Median and interquartile range [25th; 75th] of nociceptive withdrawal reflex thresholds recorded at the different time points (TP) after spinal analgesia administration.

Time point	Number of observations	Median (mA)	Interquartile range [25th; 75th] (mA)
TPBas	9	78.3	[48.6; 109,4]
TP40	7	153	[95; 153]
TPEOS	9	153	[123.1; 153]
TP240	9	153	[103.7; 153]
TP300*	7	153	[153; 153]
TP360*	7	153	[113.4; 153]
TP420	5	153	[91.7; 153]
TP480	5	140.5	[70.9; 153]

mA, milliampere. *Statistically significant difference compared to TPBas (baseline).

A statistically significant increase in NWR thresholds was found at TP300 and TP360 (*p* = 0.009 and 0.048, respectively) compared to TPBas (Figure 2).

Number of animals showing: (1) a clinically relevant increase in NWR thresholds across TPs, (2) NWR thresholds above 150 mA, (3) need of rescue analgesia, is reported in Table 2. Furthermore, trends of NWR thresholds modification across TPs per single animal is represented in Figure 3.

**Table 2:** Number of animals for each time point (TP) which showed a clinically relevant nociceptive withdrawal reflex (NWR) threshold increase [≥ 20% baseline (TPBas)], reached NWR thresholds above 150 mA, and needed rescue analgesia.

	TP40	TPEOS	TP240	TP300	TP360	TP420	TP480
NWR thresholds increase ≥ 20% baseline (TPBas)	*n* = 6/7	*n* = 8/9	*n* = 9/9	*n* = 7/7	*n* = 7/7	*n* = 4/5	*n* = 3/5
NWR thresholds > 150 mA	*n* = 4/7	*n* = 6/9	*n* = 6/9	*n* = 6/7	*n* = 5/7	*n* = 4/5	*n* = 2/5
Rescue analgesia	*n* = 0	*n* = 0	*n* = 0	*n* = 1	*n* = 0	*n* = 2	*n* = 1

Time between sedation and RM, and induction of general anesthesia and RM was 98 ± 6 and 64 ± 7 min, respectively.

**Figure 4:**
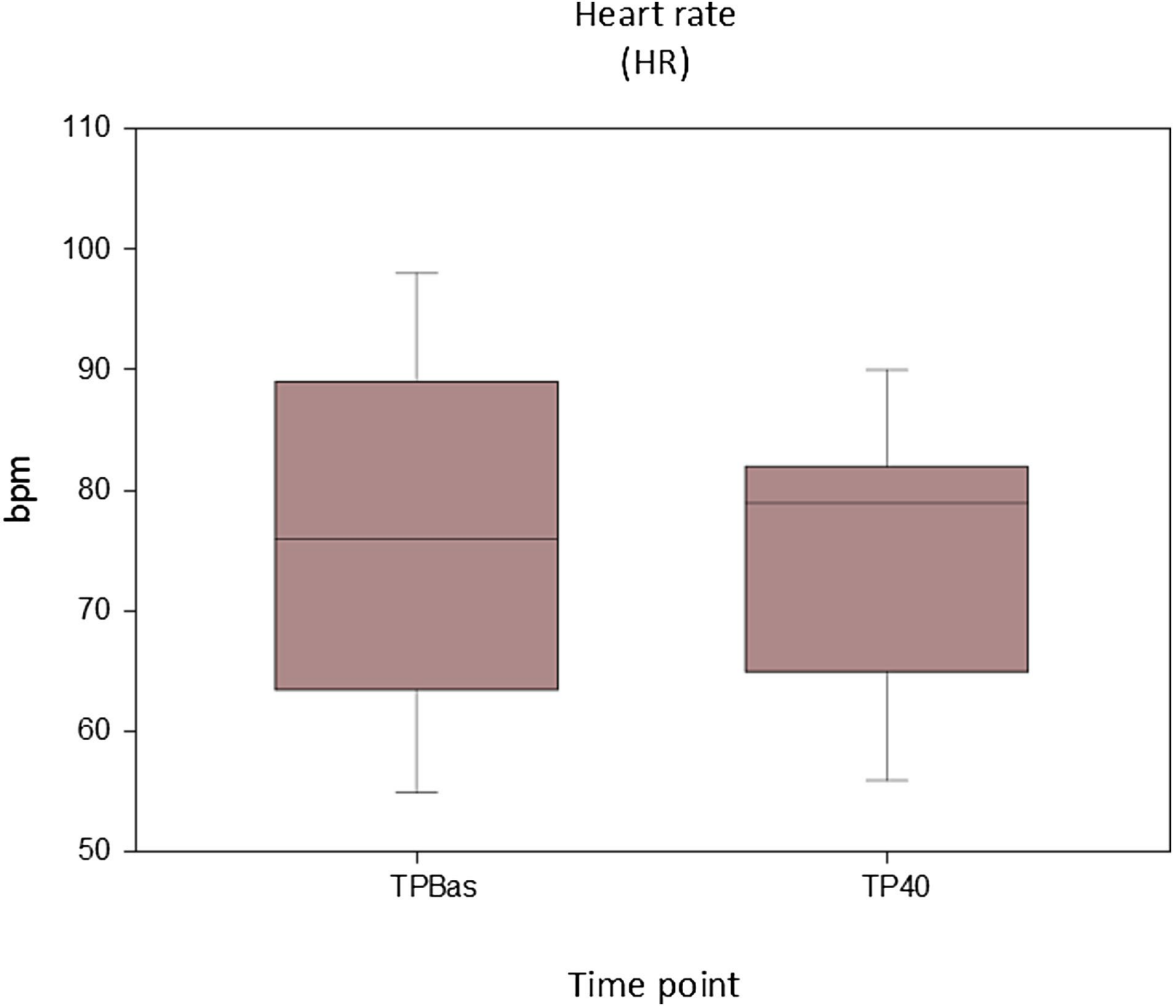
Values of heart rate recorded at baseline (TPBas) and 40 min after spinal injection (TP40). Unit: beats per minute (bpm).

**Figure 5:**
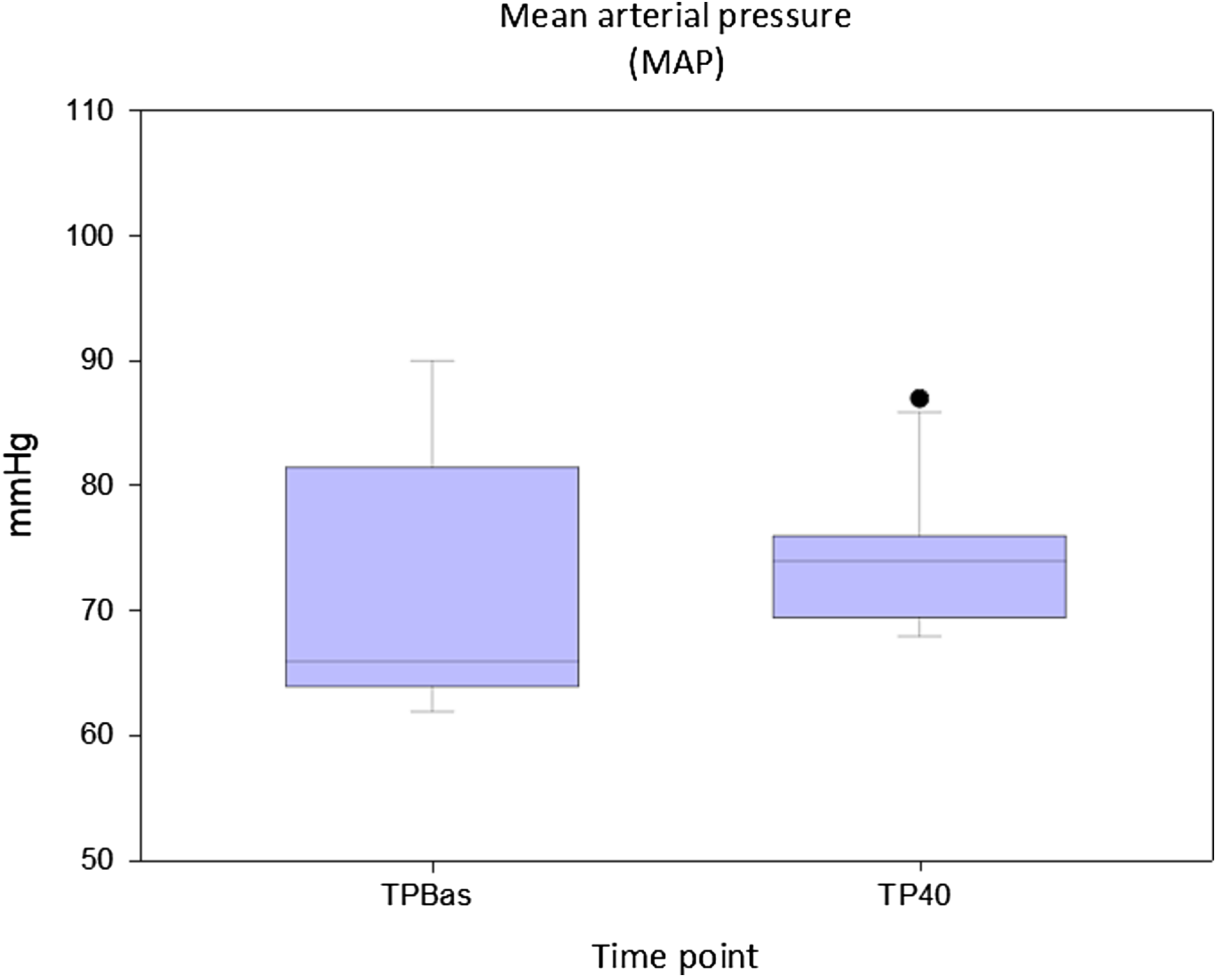
Values of mean arterial pressure at recorded at baseline (TPBas) and 40 min after spinal injection (TP40). Unit: millimeters of mercury (mmHg).

No statistically significant differences were found for both HR (*p* = 0.713) and MAP (*p* = 0.57) between TPBas and TP40 (Figures 4, 5).

Noradrenaline was needed in five animals during thoracic surgery, in one animal shortly before sternotomy, and in three after connection to the VA-ECMO. Adrenaline was needed in one animal during thoracic surgery, in three animals after connection to the VA-ECMO and in five animals after multiple weaning from the VA-ECMO.

Positive nociceptive flexor responses (claw pinching) were found only in one animal at TP300 (NWR threshold >150 mA). No presence of palpebral reflex was recorded at any TP.

## 4 Discussion

In the present study, we observed that NWR thresholds increased following administration of spinal analgesia in pigs undergoing general anesthesia for VA-ECMO. Nociceptive withdrawal reflex thresholds increased in a clinically meaningful way already at 40 min after spinal injection in all but one animal, reached their maximal values at 300 min, and remained clinically and statistically significantly higher up to 360 min. After this time point, NWR thresholds showed a decrease, the extent and the time of which were scattered. Rescue analgesia was needed in four animals. The evolution of recorded thresholds suggests that the combination of spinal morphine and ropivacaine can last on average up to 6 h. Overall, the findings of this study suggest that determination of the NWR thresholds can be of support in the evaluation of intra-operative nociception in pigs following spinal analgesia. To our knowledge, this is the first report investigating NWR threshold following spinal analgesia in this species. Previous studies in veterinary medicine have reported the use of the NWR to investigate the analgesic effect of different drugs administered systemically in both awake (22, 24, 26, 27, 29, 30) and anesthetized animals (31, 32). Furthermore, its usefulness in predicting movements in responses to nociceptive stimuli has been evaluated and demonstrated in anesthetized pigs (33) and humans (34–36).

Duration of the analgesic effect of sole ropivacaine was found to be 176.0 ± 109.0 min in a previous study in anesthetized dogs (37) and 358 [IQR 238; 538] minutes in humans (16) following spinal administration of ropivacaine 1% (dosage: 1 mg/kg) and 0.75% (dosage: 22.5 mg total), respectively. No data have been published about the analgesic duration of ropivacaine combined with morphine injected spinally in veterinary species. However, based on previous studies where this combination was used epidurally in dogs, an extension of the analgesic effect can be expected (38, 39). In the present study, following spinal analgesia, an NWR thresholds increase was found to be present until 360 min following injection in all but one animal, while in four animals lasted up to the last recording performed (480 min after spinal injection). The suggested duration should be confirmed by a higher number of observations and supported by pharmacodynamic and pharmacokinetic (PD/PK) information. Furthermore, the effects of the VA-ECMO on the PD/PK of intrathecal injected drugs is supposably negligible, but no studies have been conducted so far.

Recently, NWR thresholds in awake pigs have been reported: 7.2 [IQR 4.9; 10.5] mA (40). Similar thresholds have also been described in other species, such as horses (5.7 [IQR 5; 8.5] mA) (26) and humans (5.14 [IQR 3.03; 8.83] mA) (41). In our trial, NWR thresholds in awake animals were not assessed, and we considered as baseline the thresholds recorded in the context of a balanced anesthesia protocol. This was done to verify the hypothesis that the spinal injection of ropivacaine and morphine would increase them when general anesthesia was already at a stable depth. Our baseline values of 78.3 [IQR 48.6; 109.4] mA are not surprising in pigs receiving a protocol of balanced anesthesia. It is indeed known that the different drugs used for achieving sedation and inducing or maintaining anesthesia have an influence on the NWR (25, 32, 42, 43). Ketamine, injected in our trial in sedation, is known to be able to increase stimulation intensity required to evoke NWR in ponies (44) and a marked antinociceptive effect on high intensity nociceptive electrical stimuli has also been recognized in humans (45). In our study, when baseline thresholds were recorded, 98 ± 6 min after IM injection of ketamine, methadone, and midazolam, the plasmatic concentration of ketamine was not known. In pigs the mean half-life of 15 mg/kg ketamine injected IM was 140 min, although the duration of the anesthetic time was limited to 9–48 min in young animals, and 78–88 min in adult sows (46). Unfortunately, information regarding the onset and duration and analgesia effect is unreported (46). In humans, the association with diazepam brought about an increase of ketamine plasma concentration and a decrease of its clearance (47). We can only speculate a similar effect in pigs and assume that the baseline threshold might have been influenced by its injection. The hypnotic effect of propofol, which in our case was administered throughout the entire anesthesia, has been showed to increase NWR thresholds in humans undergoing computer-controlled propofol IV infusion at increasing dosages (median value recorded after loss of consciousness: 23.75 mA) (36). Similar trends have also been found in pigs by Mirra et al. (40), with thresholds never exceeding values of 29.4 [IQR 21.8; 35.3] mA when sole propofol was administered even though a deep anesthetic level was reached. The effect of fentanyl on the NWR was investigated in pigs undergoing isoflurane anesthesia: when used as sole analgesic, both an increase (5 μg/kg/h) and a decrease (40, 80 and 160 μg/kg/h) of the NWR area under the curve was observed (38). However, different time windows (20–100 milliseconds) to assess the reflex were selected, and recording was performed in the front limb (32).

Spinal administration of ropivacaine and morphine might lead to both sensor and motor fibers blockade, therefore elevation of the NWR thresholds can be due to both decreased peripheral sensitivity as well as motor block. The influence of the two components cannot be distinguished via the methodology used in the present study to track the thresholds. The NWR recording electrodes were positioned only at the level of the hindlimb. One could argue that high NWR thresholds could have reflected a caudal spread of the ropivacaine, accompanied by the blockade of the sensory and motor fibers innervating the hindlimb, rather than an adequate antinociception during thoracic surgery. However, in support of a cephalad spread of the spinal analgesia and therefore adequate antinociception during thoracic surgery, there are two findings. First, the high NWR thresholds recorded during and after surgery are supportive of absence of nociception during surgery. The doses of fentanyl infused during thoracic surgery are not considered antinociceptive in pigs, in which doses of 35 μg/kg/h (48) or 50 μg/kg/h (49) were needed to provide analgesia during surgical manipulations in combination to similar doses of propofol. Second, MAP and HR, before being artificially modulated, were overall stable during surgical manipulation, supporting the absence of nociceptive sympathetic stimulation as well. Further confirmation of the latter is the need of noradrenaline during surgery in six animals.

In this study, response to claw pinching was assessed regularly as a part of clinical evaluation of depth of anesthesia and recorded at each time point following determination of NWR thresholds. Motor response to claw pinching is a technique that has been frequently used to evaluate both analgesia and depth of anesthesia in pigs (19, 50, 51), however, its usefulness has been recently questioned (52). In our study, claw pinching could never be elicited but in one animal at one single time point. In this pig at 300 min after ropivacaine and morphine injection, the NWR was elicited at 150 mA, suggesting motor block and sensory block not matching in this specific circumstance. Being an isolated case, more extensive comments are not appropriate.

Cardiovascular variables, in particular MAP, has been shown to be the most reliable indicators of nociception in pigs under general anesthesia (19). In our animals, evaluation of cardiovascular parameters was reliable until TP40 following RM; 10–30 min after this time point, the VA-ECMO system was started. Stable HR and MAP were found between TPBas and TP40, reflecting adequate analgesia, and revealing no cardiovascular negative effects linked to the spinal injection. No studies investigating cardiovascular modifications due to spinal ropivacaine and morphine administration in pigs have been found, but a trial investigating the cardiovascular impact of lidocaine 2% administered spinally in experimental pigs found that its cardiovascular impact in normovolemic animals was minimal (53). Our results are in line with these findings.

This study has some limitations that needs to be acknowledged. First, no *a priori* sample size calculation was performed, and all the subjects included in the trial to evaluate VA-ECMO were recruited accordingly. Second, NWR thresholds were recorded only in the hindlimb while no records in the front limb were performed. Therefore, the effect of spinal analgesia on the sensory and motor fibers proximal to the thoracic area could not be assessed. Third, the time windows on the EMG for recognizing an NWR was set between 80 and 240 ms based on a previous investigation in pigs (33) but no clear guidelines have been established yet. Lastly, as the maximal stimulation intensity (150 mA) of the device was unmodifiable, we needed to attribute an arbitrary NWR thresholds value (153 mA), when the upper stimulation limit was overcome.

## 5 Conclusion

Nociceptive withdrawal reflex thresholds increased after spinal administration of ropivacaine and morphine. This finding supports their usefulness for evaluating antinociception following spinal analgesia in pigs, particularly in those cases in which cardiovascular variables cannot be evaluated, as during VA-ECMO. Inclusion of this monitoring technique might improve our ability to evaluate antinociception during general anesthesia following spinal analgesia. Future studies are warranted to assess the NWR thresholds’ performance in pigs receiving different systemic anesthetic/analgesic protocols.

## Data Availability

The datasets presented in this study can be found in online repositories. The names of the repository/repositories and accession number(s) can be found at: https://doi.org/10.5281/zenodo.11394587.
